# Pollution Flashover Characteristics of Hydrophilic/Hydrophobic Alternating Surfaces for Insulator Hybridization

**DOI:** 10.3390/polym18080904

**Published:** 2026-04-08

**Authors:** Bo Tao, Li Cheng, Yi Gong, Haoming Bao, Ruijin Liao

**Affiliations:** State Key Laboratory of Power Transmission Equipment Technology, School of Electrical Engineering, Chongqing University, Chongqing 400044, China; taobo@stu.cqu.edu.cn (B.T.); yi_gong2003@163.com (Y.G.); cy7130481@163.com (H.B.); rjliao@cqu.edu.cn (R.L.)

**Keywords:** hybrid insulator, pollution flashover, hydrophobicity, dry band, multi-arcs

## Abstract

With the growing trend toward insulator hybridization, higher requirements are imposed on the synergistic improvement of interfacial durability and pollution flashover performance. Machining annular grooves at the green-body stage and embedding silicone rubber enables the construction of an embedded structure with improved durability, forming hydrophilic/hydrophobic alternating surfaces. However, the outdoor insulation characteristics of such hybrid surfaces remain insufficiently investigated, and their engineering feasibility requires further validation. In this study, a series of hydrophilic/hydrophobic alternating surfaces were fabricated, and artificial pollution tests were conducted. The results show that the AC pollution flashover voltage exhibits a saturated increasing trend as the hydrophobic interfaces become more dispersed. When twenty 4 mm wide hydrophobic interfaces were distributed along a 16 cm creepage distance, the flashover voltage was 12.4% higher than that of a fully hydrophobic surface. These results indicate that appropriate design of hydrophobic interface distribution can achieve excellent pollution flashover performance even at relatively low hydrophobic coverage (≤50%). High-speed imaging combined with infrared thermography reveals the discharge mechanism governed by hydrophobic interface distribution from an electro–thermal coupling perspective. The coexistence of multiple dry bands induced by discrete hydrophobic interfaces is identified as the key factor enhancing flashover withstand capability. A static pollution flashover model was established to quantitatively estimate the AC flashover voltage, confirming the external insulation feasibility of the embedded hybrid concept.

## 1. Introduction

**Figure 1:**
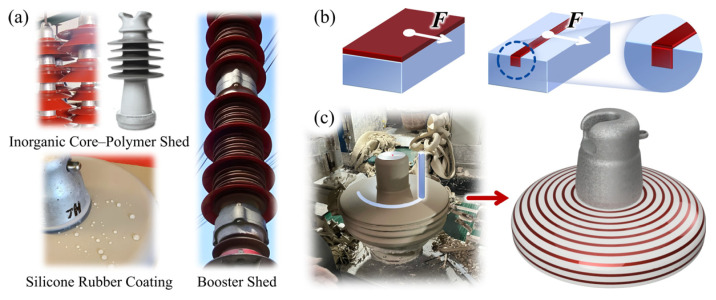
(**a**) Existing types of hybrid insulators; (**b**) Advantages of the embedded interfacial bonding structure; (**c**) Fabrica-tion of the embedded hybrid insulator.

Insulators can generally be classified into two categories according to material type [1,2]: (1) ceramic and glass insulators, which exhibit excellent aging resistance and high mechanical strength but possess hydrophilic surfaces; and (2) composite insulators, which provide hydrophobic surfaces and superior electrical performance, but comparatively lower aging resistance and mechanical strength. To combine the mechanical reliability of inorganic materials with the hydrophobic performance of polymers, hybrid solutions have gradually been adopted in engineering practice, including inorganic core–polymer sheath structures [3], silicone rubber coatings [2,4], and additional booster sheds [5], as shown in Figure 1a. As a result, outdoor insulation structures are increasingly evolving toward hybrid configurations. However, in existing hybrid designs, silicone rubber is typically bonded to ceramic or glass substrates in the form of surface coatings or external sheaths. The long-term reliability of these structures largely depends on interfacial chemical adhesion. Under prolonged environmental stresses [6,7,8], interfacial aging can lead to sheath debonding or coating delamination [9,10,11], which becomes a critical bottleneck limiting service life. For example, the typical service lifetime of room temperature vulcanized (RTV) silicone rubber anti-pollution coatings is only 8–12 years [2,12,13].

In recent years, a strategy based on modifying interfacial geometry to enhance the bonding reliability of heterogeneous materials has attracted increasing attention, namely transforming conventional two-dimensional interfaces into embedded three-dimensional interfacial structures [14]. This approach has been successfully validated in various fields, including polymer–metal bonding [15,16,17], soft tissue adhesion [18], and condenser design [19], where it can achieve an order-of-magnitude improvement in adhesion strength [16,18,19]. In this study, this concept is introduced into the field of hybrid insulators by embedding silicone rubber into pre-fabricated grooves of the substrate to form an embedded structure. Compared with traditional surface coating or encapsulation methods, the embedded structure offers two main advantages (Figure 1b). First, it significantly increases the effective bonding area [20]. In addition to the bottom interface, the large-area sidewall interfaces also contribute to load transfer, thereby enhancing the overall structural strength. Second, the embedded structure introduces a mechanical interlocking effect. Under shear stress (*F*), failure in conventional coating structures is primarily governed by the shear fracture of interfacial chemical bonds. In contrast, for the embedded structure, the shear load is redistributed between the bottom and sidewall interfaces, with the sidewalls of the substrate subjected to compressive stresses. This transforms the failure mode from single-interface debonding into a coupled interface–substrate failure mechanism [20], thereby significantly improving resistance to interfacial peel-off.

It should be emphasized that the design of embedded structures must consider manufacturing feasibility. In accordance with the production process of ceramic insulators [21], concentric annular grooves can be machined at the green-body stage using modified tools (Figure 1c). This approach is easy to implement, highly compatible with existing manufacturing systems, and preserves the integrity of the glaze layer. By embedding silicone rubber into these annular grooves, a hydrophilic/hydrophobic alternating surface can be formed. However, the outdoor insulation characteristics of such complex surfaces remain insufficiently studied. Therefore, prior to engineering implementation, it is necessary to verify their outdoor insulation feasibility.

For hydrophilic/hydrophobic alternating surfaces, the surface conductivity of the pollution layer on hydrophobic regions is significantly lower than that on hydrophilic regions after wetting [22]. This conductivity contrast allows the structure to be approximately regarded as a pollution layer with periodically distributed artificial high-resistance strips [23,24]. Based on this equivalence, the analytical framework developed for dry-band number, width, and location can be adopted to evaluate their influence on outdoor insulation performance. Dry bands act as potential barriers within the conductive pollution layer. They induce local electric field enhancement and trigger surface air breakdown, leading to the formation of dry-band arcs [23,25,26]. Compared with metal electrodes, dry-band arcs in series with electrolyte liquid films exhibit an additional voltage drop of approximately 0.85 kV [1]. In the Obenaus model, this arc-root voltage drop contributes to an increase in the withstand voltage. Specifically, doubling the dry-band width increases the AC pollution flashover voltage by 1.2–7.8% [27,28]. When the number of dry bands increases from one to three, the AC pollution flashover voltage rises by 9.5–10.6% [28,29]. As the dry-band number increases, the surface potential distribution of the polluted layer evolves from globally highly non-uniform to globally uniform with local non-uniformity. Accordingly, the role of dry bands shifts from a simple barrier effect to a potential homogenization effect [30]. Therefore, under multi-dry-band conditions, the flashover voltage is generally higher than that under uniform pollution or single dry-band configurations. It is worth noting that previous studies [31,32] have extended the Obenaus model by introducing a source term associated with arc root resistance. This enables the model to describe pollution flashover on hydrophobic and superhydrophobic surfaces. From a physical perspective, such extended Obenaus models reflect the segmented development of arcs on hydrophobic surfaces [1,33]. These findings suggest that hydrophilic/hydrophobic alternating surfaces may similarly induce multi-arc formation through multiple dry bands, thereby maintaining a relatively high flashover voltage.

In summary, this study aims to validate the feasibility of the embedded hybrid concept. A series of hydrophilic/hydrophobic alternating surfaces with different numbers, widths, and spacings of hydrophobic interfaces were designed and fabricated. Artificial pollution flashover tests were conducted, and the discharge process was jointly observed using high-speed imaging and infrared thermography. Based on these investigations, the pollution flashover characteristics of the alternating surfaces and the mechanisms governing their discharge behavior were clarified. A static pollution flashover model applicable to hybrid surfaces was further established, enabling quantitative estimation of the flashover voltage. The results provide theoretical support for the design and manufacturing of embedded hybrid insulators and contribute to the understanding of pollution flashover behavior on complex heterogeneous surfaces.

## 2. Methods

### 2.1. Sample Preparation

**Figure 2:**
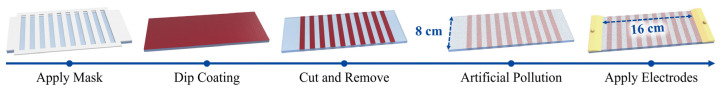
Preparation process of the hydrophilic/hydrophobic alternating surfaces.

A hydrophilic glass plate (200 × 80 × 5 mm) was used as the substrate. Considering the engineering applicability and self-leveling characteristics of RTV coatings, PRTV (RTV–II, Hebei Guigu, China) was applied to form the hydrophobic interfaces. The sample preparation process is illustrated in Figure 2. Transparent adhesive tape cut using a stainless-steel mesh was employed as a mask to control the distribution of hydrophilic and hydrophobic regions on the alternating surface. The thickness of the hydrophobic coating was maintained at 0.4–0.5 mm, and the samples were cured at 25 °C and 60% relative humidity (RH) for 72 h.

Sodium chloride and diatomite were used as artificial pollution materials. The non-soluble deposit density (NSDD) and equivalent salt deposit density (ESDD) were set to 1.0 mg/cm^2^ and 0.1 mg/cm^2^, respectively. According to the standard [34], the samples were polluted using the solid layer method. The deviation in pollution severity between the hydrophilic and hydrophobic regions was controlled within 8%. For samples containing hydrophobic interfaces, after pollution the specimens were kept in a constant temperature and humidity chamber at 25 ± 1 °C and 50 ± 3% RH for 48 h to allow hydrophobicity transfer.

### 2.2. Test Platform

**Figure 3:**
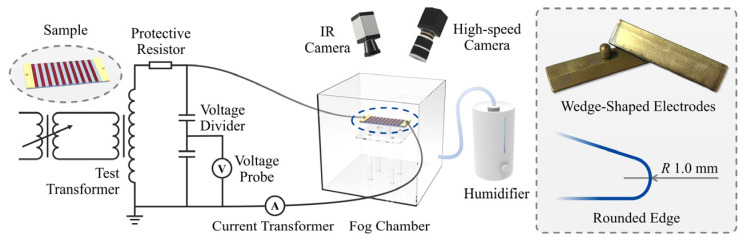
Schematic diagram of the artificial pollution test platform.

The artificial pollution test platform consisted of a high-voltage power supply, a fog chamber, and a signal acquisition system, as shown in Figure 3. The power supply was rated at 50 kV/50 kVA and operated at 50 Hz, with a protective resistor of 5 kΩ. The voltage regulator had a rated current of 119 A. The inner dimensions of the fog chamber were 500 × 500 × 500 mm. A clean fog environment was generated using an ultrasonic humidifier with a mist output rate of 800 mL/h. The droplet diameter ranged from 1 to 10 μm, and the fog was introduced from the bottom of the chamber. Copper wedge-shaped electrodes (85 × 20 × 4 mm) were used. To minimize electric field distortion, all electrode edges were rounded with a radius of 1 mm. The high-voltage and ground electrodes were symmetrically arranged in contact with the pollution layer surface of the sample. The effective creepage distance of the specimen was 160 mm.

The signal acquisition system consisted of an electrical measurement module and an imaging module. Voltage and leakage current signals were measured using a high-voltage differential probe (attenuation ratio: 2000:1) and a current transformer (5 V/5 A), respectively. The signals were synchronously recorded by a data acquisition card (NI USB-6211) at a sampling rate of 40 kS/s and visualized and stored in real time using MATLAB 2024b.

The up-and-down voltage method [34] was adopted for the pollution flashover tests. The secondary-side set current was 20 A, and the voltage was adjusted in steps of 1 kV. Each test condition was repeated at least ten times. Since this method requires wetting under energized conditions with the fog chamber sealed, direct observation of the discharge process was difficult. Therefore, the even-rising method was employed during discharge imaging, with the set current increased to 40 A.

The imaging module included high-speed visible imaging and infrared thermography. A high-speed camera (M220, Revealer, Chongqing, China) was used to record arc development, with a frame rate of 4065 fps, an exposure time of 241 μs, an aperture of *f* 2.0, and a resolution of 1152 × 532 pixels. In addition, an infrared thermal camera (HiNet-640, IRSV, Beijing, China) was used to monitor the temperature distribution of the pollution layer on the sample surface. The camera had a spatial resolution of 640 × 512 pixels, a frame rate of 30 fps, a temperature measurement range of −20 °C to +550 °C, and an accuracy of ±2 °C.

## 3. Results

For the hydrophilic/hydrophobic alternating surfaces, the area fraction of hydrophobic interfaces (*s*) was fixed at 50%, and the hydrophobic interfaces were uniformly spaced. On this basis, alternating surface samples with different numbers of hydrophobic interfaces (*n*_phobic_) were constructed by varying the width (*w*) of each individual hydrophobic interface. To quantitatively characterize the degree of alternation, an alternating density (*ρ*) was defined as the ratio of the total number of hydrophilic/hydrophobic interfaces to the creepage distance (*l* = 16 cm):

(1) $$\rho =(2\cdot n_{\mathrm{phobic}}-1)/l$$

The sample configurations and corresponding parameters used in this study are summarized in Table 1.

**Figure 4:**
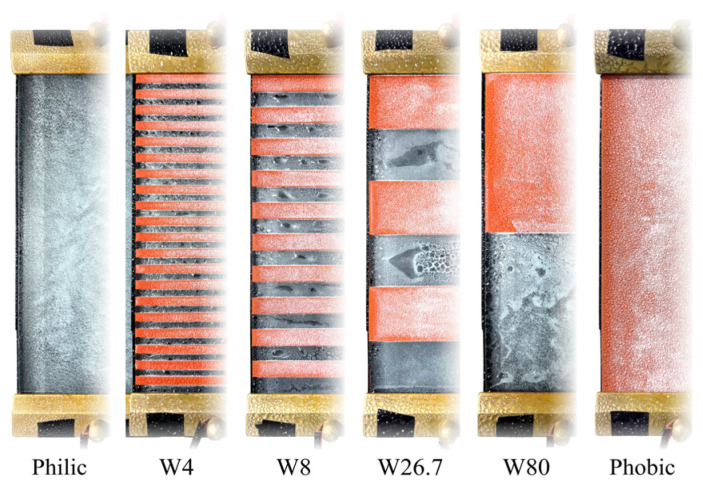
Wetting morphology of the samples.

According to the standard [34], the samples were subjected to saturated wetting, with the criterion defined as the pollution layer conductivity reaching its maximum value, which occurred after approximately 15–18 min. The morphology of the pollution layer under saturated wetting conditions for alternating surfaces with different alternating densities (*ρ*) is shown in Figure 4. For the purely hydrophilic sample, the pollution layer formed a continuous water film. Owing to the hydrophobicity transfer property of silicone rubber [35], the purely hydrophobic sample exhibited discrete water droplets on the pollution layer surface. In contrast, the hydrophilic/hydrophobic alternating surfaces exhibited characteristics of both cases. The wetted morphology consisted of periodic alternations of continuous water films and discrete droplets along the creepage direction. As *ρ* increased, the number of hydrophobic interfaces increased accordingly. The originally continuous water film was repeatedly segmented, and its continuity decreased significantly.

**Figure 5:**
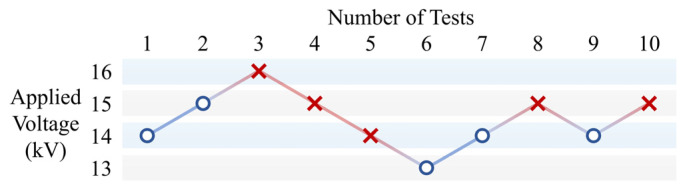
Up-and-down voltage test process of the W4 sample, where “o” denotes withstand and “×” denotes flashover.

**Figure 6:**
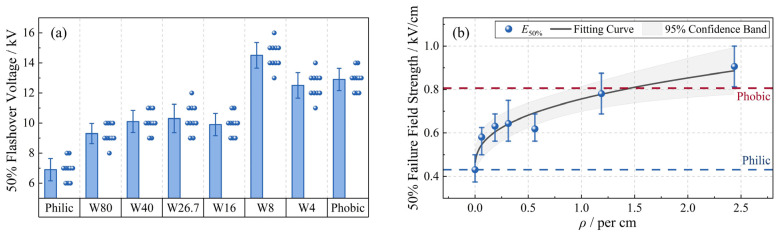
(**a**) The 50% flashover voltage of the samples. (**b**) Variation in the 50% failure electric field strength with alternating density (*ρ*).

The up-and-down voltage test process for the W4 sample is shown in Figure 5. The 50% pollution flashover voltage, *U*_50%_, was calculated using the following equation:

(2) $$U_{50\%}=\left(\sum \left(n_{i}\times U_{i}\right)\right)/N$$

where *U_i_* is a specific applied voltage level, *n_i_* is the number of tests conducted at the same voltage level, and *N* is the total number of valid tests. The *U*_50%_ values of all samples are shown in Figure 6a.

Furthermore, the 50% failure field strength, *E*_50%_, was defined as

(3) $$E_{50\%}=U_{50\%}/l$$

The relationship between *E*_50%_ and the alternating density (*ρ*) is presented in Figure 6b. As *ρ* increased, *E*_50%_ exhibited a clear monotonic increase with a saturation tendency. This trend was fitted using a power-law function:

(4) $$E_{50\%}(\rho )=0.43+0.33\rho^{0.38},\;\;\;\;\rho \in (0,2.44)$$

The coefficient of determination was *R*^2^ = 0.9. Compared with the purely hydrophilic sample, the introduction of hydrophobic interfaces significantly enhanced the failure field strength of the alternating surfaces. As the hydrophobic interfaces became more dispersed (i.e., with increasing *ρ*), the insulation strength further increased and even exceeded that of the purely hydrophobic sample. These results demonstrate that by regulating the distribution of hydrophobic interfaces on hydrophilic/hydrophobic alternating surfaces—i.e., by adjusting *ρ*—the pollution flashover voltage can be maintained or even enhanced under a limited hydrophobic area fraction (≤50%).

To clarify why the pollution flashover voltage did not decrease under a low hydrophobic area fraction and to further reveal the intrinsic mechanism underlying the saturation increase in flashover voltage with alternating density, discharge observations were conducted using the even-rising method. During the tests, the samples were fully wetted prior to energization until the leakage current reached its maximum value. The flashover process was then synchronously recorded using high-speed visible imaging and infrared thermography.

**Figure 7:**
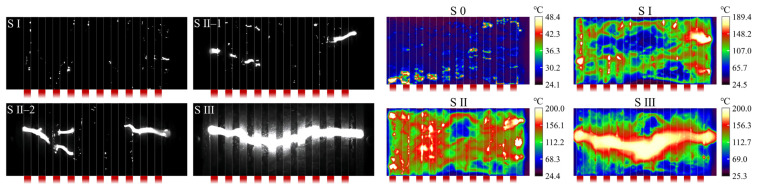
High-speed visible and infrared images of the pollution flashover process for the W8 sample. Red strips denote the locations of the hydrophobic interfaces.

The pollution flashover processes of the purely hydrophilic and hydrophobic samples are shown in Figures S1 and S2, respectively, while the visible–infrared discharge process of the W8 sample is presented in Figure 7. Based on the arc development characteristics, the flashover process was divided into three stages: Stage I: widespread spot discharges; Stage II: segmented arcs with restricted merging; Stage III: complete flashover.

In Stage I, spot discharges were primarily concentrated at the hydrophilic/hydrophobic interfaces. Some discharges even formed a linear pattern perpendicular to the creepage direction, coinciding with the interface. In Stage II, the merging of adjacent arcs was restricted, leading to the formation of millimeter-scale short arcs at the macroscopic level. For the W8 sample, the discharge channel in this stage consisted of five to six short arcs. This phenomenon resembled the multi-arcs effect observed on purely hydrophobic surfaces (Figure S2).

**Figure 8:**
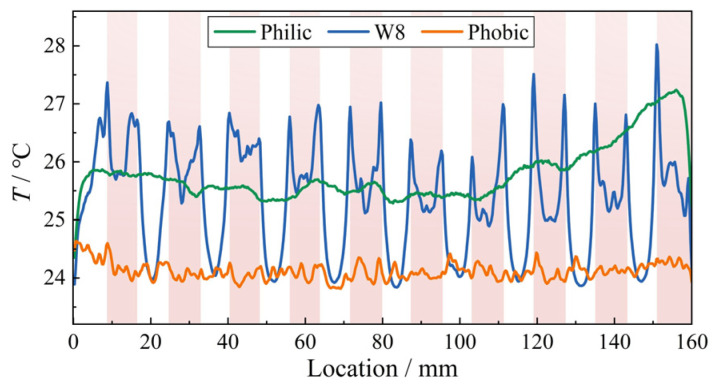
Mean pollution layer temperature along the creepage path for the Philic, W8, and Phobic samples, where the red regions indicate the hydrophobic interfaces of the W8 sample.

Further analysis was conducted based on infrared thermography. At the initial withstand stage (Stage 0), Joule heating was mainly concentrated in the hydrophobic interface regions. After entering Stages I and II, numerous hotspots appeared at the hydrophilic/hydrophobic interfaces. Their spatial distribution closely coincided with the locations of spot discharges observed in the high-speed images. To quantitatively evaluate the temperature difference between regions with different wettability, the temperature contour map at the early Stage I was extracted. The temperature distribution was averaged along the direction perpendicular to the creepage path, yielding the mean temperature profile along the creepage direction (Figure 8). The results show that, for the W8 sample, the average temperature of the pollution layer above the hydrophobic interfaces is more than 2 °C higher than that above the hydrophilic regions. In addition, pronounced local temperature peaks are observed at the hydrophilic/hydrophobic interfaces, corresponding to the spikes in the mean temperature profile, whereas no such peaks appear in the temperature distribution curves of homogeneous surfaces. With increasing applied voltage and continuous Joule heating, the temperature in the interface regions rose rapidly. The local maximum temperature reached up to 267 °C, far exceeding the boiling point of water.

Based on these observations, two key discharge characteristics of the hydrophilic/hydrophobic alternating surface were identified: (1) widespread and dense spot discharges at the interfaces, and (2) the multi-arcs effect.

**Figure 9:**
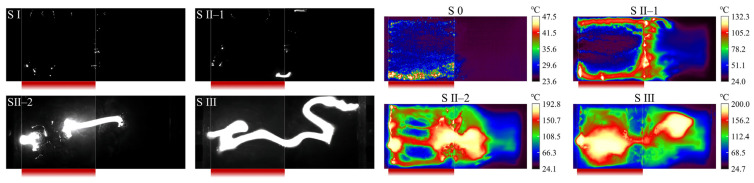
High-speed and infrared images of the pollution flashover process for the W80 sample. Red strips denote the locations of the hydrophobic interfaces.

To verify the consistency of the above discharge phenomena under different alternating densities, the W80 sample was analyzed as an extreme case. Its high-speed visible and infrared flashover process is shown in Figure 9. In Stage 0, Joule heating was almost entirely concentrated in the hydrophobic interface region on the left side of the sample. The thermal distribution corresponded to corona discharges occurring between discrete water droplets. In Stages I and II, abundant spot discharges were again observed at the hydrophilic/hydrophobic interface on the W80 surface. The discharges were mainly concentrated at the central interface and at the contact region between the hydrophobic interface and the electrode. Meanwhile, arc development tended to initiate and propagate around the interface, exhibiting discharge characteristics consistent with those of the W8 sample. Notably, since the W80 sample contained only one hydrophilic/hydrophobic interface, only two arc segments were observed in Stage II, which was significantly fewer than in the W8 sample. These results indicate that discharge characteristic I—widespread spot discharges at the interfaces—is universal for alternating surfaces, whereas discharge characteristic II—the multi-arcs effect—is strongly influenced by the alternating density.

## 4. Discussion

In the previous section, the pollution flashover characteristics of hydrophilic/hydrophobic alternating surfaces were systematically investigated, and two representative discharge features were identified. This section focuses on the mechanisms governing these discharge features and on how the discharge behavior is controlled by the distribution of hydrophobic interfaces.

### 4.1. Mechanism of Widespread Spot Discharges at Hydrophilic/Hydrophobic Interfaces

**Figure 10:**
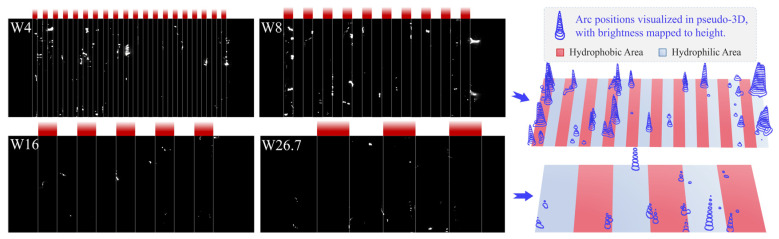
Representative Stage I high-speed images for different values of *ρ*, with pseudo-3D visualization of arc locations based on brightness information.

High-speed images captured at representative moments in Stage I for the W4, W8, W16, and W26.7 samples are shown in Figure 10. Despite differences in alternating density, the same characteristic phenomenon was consistently observed. Widespread spot discharges at the interfaces can be clearly identified from the pseudo-3D visualization. This feature is particularly pronounced for the W4 and W8 samples, where spot discharges occasionally form band-like structures along the hydrophilic/hydrophobic interfaces, extending perpendicular to the creepage direction. To elucidate the formation mechanism of these interface spot discharges, the analysis is conducted based on the conductivity distribution of the pollution layer on the alternating surface.

**Figure 11:**
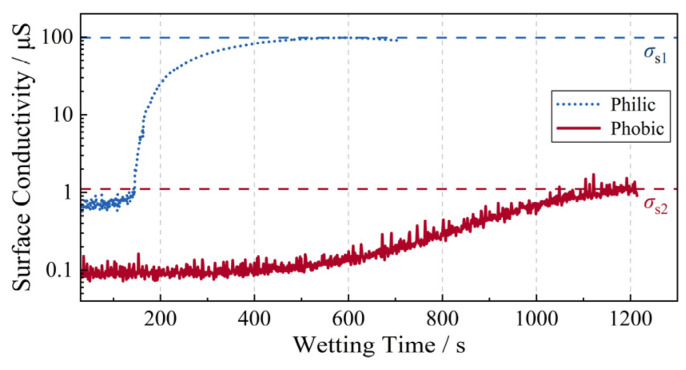
Surface conductivity of the pollution layer during the wetting process for the Philic and Phobic samples.

First, the surface conductivity of the pollution layer over the hydrophilic regions is denoted as *σ*_s1_, while that over the hydrophobic regions is denoted as *σ*_s2_. During the wetting process, the variation in the surface conductivity of the pollution layer is monitored in real time according to the following equation, based on the wetting voltage (*U*_W*i*_) and the peak value of the leakage current (max(*I*_W*i*_)) [34]:

(5) $$\sigma_{si}=\frac{f\cdot \max(I_{Wi})}{\sqrt{2}U_{Wi}}\cdot [1-0.022(T-20)]$$

where *T* is the fog chamber temperature, and *f* is the shape factor of the sample, which is taken as 2 in this study. The obtained variations in *σ*_s1_ and *σ*_s2_ are shown in Figure 11. It can be observed that, under saturated wetting conditions, *σ*_s1_ and *σ*_s2_ are 98.31 μS and 1.11 μS, respectively. Therefore, *σ*_s1_ is approximately two orders of magnitude higher than *σ*_s2_, i.e., *σ*_s1_/*σ*_s2_ ≈ 100. This result is consistent with previous studies [22,33,36].

As a result, along the creepage direction the alternating surface can be equivalently regarded as a series non-uniform impedance distribution. Under the applied voltage *U*, the majority of the voltage drop occurs across the hydrophobic regions, leading to local electric stress concentration. Under this assumption, the electric stresses on the hydrophilic and hydrophobic regions, denoted as *E*_1_ and *E*_2_, respectively, can be expressed as

(6) $$E_{1}=\frac{U}{l(s+(\sigma_{s1}/\sigma_{s2})\cdot (1-s))}\approx \frac{U}{100\cdot l\cdot (1-s)}$$

(7) $$E_{2}=\frac{U}{l((1-s)+s/(\sigma_{s1}/\sigma_{s2}))}\approx \frac{U}{l\cdot (1-s)}$$

where *s* is the fraction of hydrophobic area, ranging from 0 to 1. It follows that the electric stress on the hydrophobic interfaces is significantly higher than that on the hydrophilic regions.

**Figure 12:**
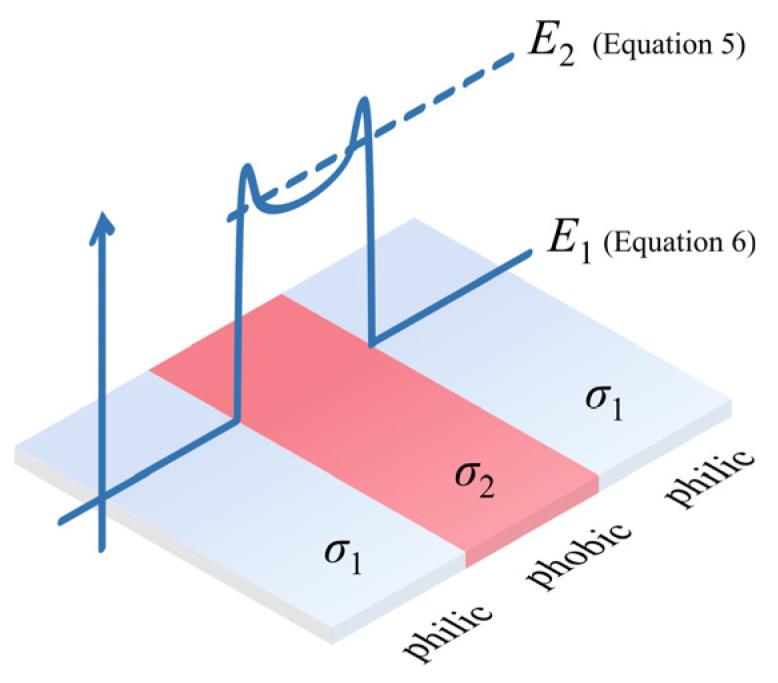
Schematic of electric stress distribution on a hydrophilic/hydrophobic alternating surface.

Therefore, for a “hydrophilic–hydrophobic–hydrophilic” alternating configuration (Figure 12), the hydrophobic region can be abstracted as a high-resistance section sandwiched between two conductive electrolyte liquid films. Considering edge effects, a triple-junction point is formed at the intersection of the hydrophilic region, hydrophobic region, and air—that is, at the hydrophilic/hydrophobic interface. At this location, the local electric field gradient (*E*_inter_) becomes distorted. When it exceeds the surface breakdown strength of air (*E*_air_), i.e., when the following condition is satisfied:

(8) $$E_{\mathrm{int}\mathrm{er}}>E_{\mathrm{air}}$$

surface air breakdown occurs. Macroscopically, this manifests as widespread and dense spot discharges at the hydrophilic/hydrophobic interfaces.

### 4.2. Mechanism of the Multi-Arcs Effect

The multi-arcs effect is another key discharge characteristic of hydrophilic/hydrophobic alternating surfaces. Importantly, this effect does not arise from a continuous arc being blocked or segmented during propagation. Instead, the discharge initiates as multiple discrete millimeter-scale short arcs. A central question therefore concerns the origin of these arc segments: do they initiate above the hydrophilic regions or the hydrophobic interfaces?

**Figure 13:**
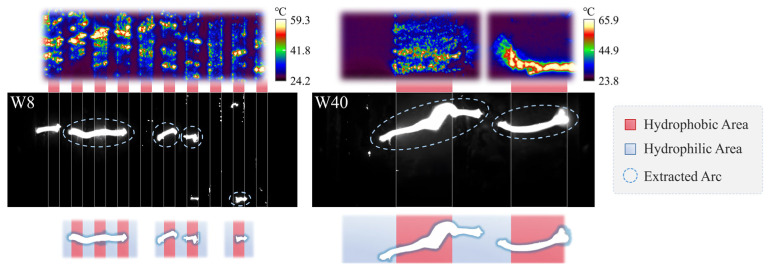
Arcs observed above hydrophobic interfaces during flashover of W8 and W40 samples.

To address this issue, a combined analysis of high-speed imaging and infrared thermography was performed for the W8 and W40 samples (Figure 13). During the initial withstand stage, thermal hotspots were predominantly concentrated in the pollution layer above the hydrophobic interfaces. In Stage II, arc segments bridging across the hydrophobic interfaces were consistently observed. These observations indicate that the multi-arcs effect originates from bridging arcs formed above the hydrophobic interfaces. In this process, the hydrophobic interfaces function analogously to artificial dry bands. Owing to the intentionally introduced periodic distribution of multiple hydrophobic interfaces, several dry bands can form simultaneously on the alternating surface. Each dry band sustains a stable dry-band arc, resulting in multiple arc segments along the creepage path. Macroscopically, this manifests as the multi-arcs effect. Based on these observations, the intrinsic mechanism by which hydrophobic interfaces govern dry-band formation and induce the multi-arcs effect is further elucidated from the perspective of electro–thermal coupling within the pollution layer.

Similarly, the dominance of hydrophobic interfaces in dry-band formation also originates from the non-uniform conductivity distribution of the pollution layer. This conductivity contrast leads to localized Joule heating, which subsequently induces non-uniform moisture evaporation within the pollution layer. Previous studies [25,37] indicate that when the local Joule heating power in a region (*P*′) exceeds the minimum power required for dry-band initiation (*P*_min_), and its growth rate remains higher than that of the surrounding regions, non-uniform evaporation occurs and a dry band forms in that region. The corresponding criterion can be expressed as

(9) $$\left\{\begin{array}{l} P^{\prime }>P>P_{\min}\\ dP^{\prime }/dt>dP/dt \end{array}\right.$$

Once this criterion is satisfied, a positive feedback mechanism is established. The accelerated evaporation further intensifies local heating, ultimately leading to irreversible dry-band formation.

For hydrophilic/hydrophobic alternating surfaces, the above non-uniform evaporation criterion remains applicable. The surface electric field (*E*), Joule heating power (*P*), and current density (*J*) of the pollution layer are related to the surface conductivity (*σ*_s_) through the following constitutive relationships:

(10) $$\left\{\begin{array}{l} E=J/\sigma_{s}\\ P=J^{2}/\;\sigma_{s} \end{array}\right.$$

In addition, *σ*_s_ is related to the electric field *E* as follows [37]:

(11) $$\sigma_{s}(E)=\sigma_{s}(0)/(1+\sigma_{s}(0)AE^{2})$$

Substituting Equation (10) into Equation (11) yields

(12) $$\sigma_{s}(E)=\sigma_{s}(0)(1-AP)$$

At the initial stage, when the applied voltage is first imposed on the alternating surface, the Joule heating power above the hydrophilic region (*P*_1_) and that above the hydrophobic region (*P*_2_) are both very small. Under this condition, 1 − *AP* ≈ 1, and thus

(13) $$\frac{P_{2}(0)}{P_{1}(0)}\approx \frac{\sigma_{s1}(0)}{\sigma_{s2}(0)}>1$$

This relationship indicates that even at the early withstand stage, the Joule heating power in the pollution layer above the hydrophobic interface is already significantly higher than that above the hydrophilic region. To further examine the temporal evolution of this difference, the relationship between *P*_1_ and *P*_2_ with increasing energization time can be expressed as

(14) $$\frac{P_{2}}{P_{1}}=\frac{\sigma_{s1}(0)(1-AP_{1})}{\sigma_{s2}(0)(1-AP_{2})}\gg 1$$

Since *P*_2_(0) > *P*_1_(0), the subtractive term in the denominator grows much faster than the numerator. As a result, the initial power difference—on the order of two magnitudes—is progressively amplified, ultimately leading to *P*_2_ >> *P*_1_. Therefore, throughout the withstand process, the first criterion for dry-band initiation remains satisfied. Differentiating Equation (14) with respect to *P*_1_ yields

(15) $$\frac{dP_{2}}{dP_{1}}=\frac{dP_{2}/dt}{dP_{1}/dt}=\frac{\sigma_{s1}(0)(1-2A_{1}P_{1})}{\sigma_{s2}(0)(1-2A_{2}P_{2})}\gg 1$$

**Figure 14:**
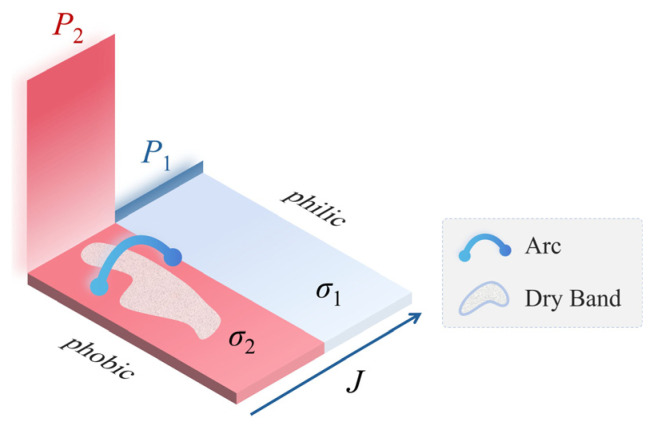
Schematic of Joule heating power distribution on a hydrophilic/hydrophobic alternating surface.

The result shows that the growth rate of Joule heating power above the hydrophobic interface is significantly higher than that above the hydrophilic region. This establishes a pronounced positive feedback mechanism: higher Joule heating accelerates moisture evaporation, which further reduces the local conductivity and enhances electric field concentration. Consequently, dry bands preferentially form above the hydrophobic interfaces, as schematically illustrated in Figure 14.

Given the periodic distribution of hydrophobic interfaces on the alternating surface, multiple dry bands can form simultaneously. This ultimately induces a stable multi-arcs discharge configuration at the macroscopic level.

## 5. Static Pollution Flashover Model for Alternating Surfaces

Based on the mechanistic analysis of discharge characteristics presented in the previous section, a static pollution flashover model for the alternating surface is further established. To construct the model, it is first necessary to clarify the composition of the multi-arcs discharge channel. Therefore, high-speed images captured immediately prior to flashover (late Stage II to early Stage III) are analyzed to determine the merging process of arc segments on the alternating surface.

**Figure 15:**
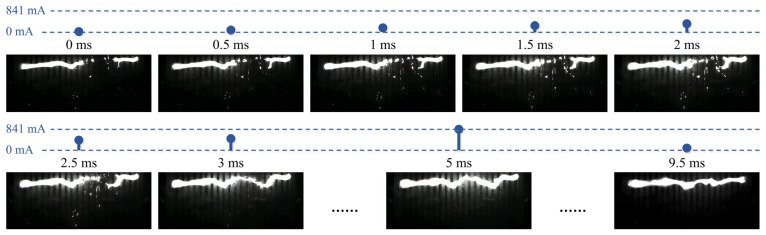
High-speed sequence of arc development on the hydrophilic/hydrophobic alternating surface before flashover, with the blue curve indicating the leakage current magnitude in the corresponding half-cycle.

Taking the W4 sample as an example, the arc development within half a cycle prior to flashover is shown in Figure 15. This half-cycle corresponds to the positive half-wave, with the left electrode at high potential and the right end of the arc acting as the cathode. A characteristic “claw” structure is observed at the cathode root [38,39]. On the hydrophilic/hydrophobic alternating surface, multiple millimeter-scale short arcs are distributed between the two main arcs. Closer examination shows that these short arcs predominantly occur at the hydrophobic interfaces, while they are electrically connected through the residual pollution layer over the hydrophilic regions. Specifically, four arc-merging events across the residual pollution layer in the hydrophilic regions occur within 1–3 ms. When the current reaches its peak value of 841 mA (approximately 5 ms), the discharge channel becomes fully bridged, ultimately leading to flashover.

In comparison, although both the alternating surface and the purely hydrophobic surface (Figure S4) exhibit multi-arcs characteristics, their discharge channel compositions are fundamentally different. For the hydrophobic surface, multi-arcs originate from corona discharges at the ends of elongated filamentary water channels [40,41], and the discharge path consists of arc segments interspersed with water filaments. In contrast, for the alternating surface, multi-arcs originate from multiple dry-band arcs formed above the hydrophobic interfaces, and these arc segments are connected in series through the residual pollution layer on the hydrophilic regions.

Based on the clarified discharge channel compositions of the three surface types prior to flashover, static pollution flashover models for hydrophilic, hydrophobic, and alternating surfaces can be established according to the Obenaus theory [1,40,42]:

(16) $$\left\{\begin{array}{l} U_{\mathrm{philic}}=n_{\mathrm{philic}}(U_{a}+U_{c})+xAI^{-\alpha }+R_{\mathrm{rpl}}(x)I\\ U_{\mathrm{phobic}}=n_{\mathrm{phobic}}(U_{a}+U_{c})+xAI^{-\alpha }+R_{\mathrm{wr}}(x)I\\ U_{\mathrm{as}}=n_{\mathrm{as}}(U_{a}+U_{c})+xAI^{-\alpha }+R_{\mathrm{rpl}}(x)I \end{array}\right.$$

where *U*_#_ denotes the applied voltage, *I* is the leakage current, and *x* is the total arc length. *U*_a_ and *U*_c_ represent the anode and cathode voltage drops of the arc, respectively, with *U*_a_ + *U*_c_ = 0.84 kV. The term *n*_#_ corresponds to the number of arc segments for different surface types. The arc voltage drop is expressed as *xAI*^−*α*^. *R*_rpl_(*x*) and *R*_wr_(*x*) denote the resistance of the residual pollution layer and the water ribbon, respectively.

**Figure 16:**
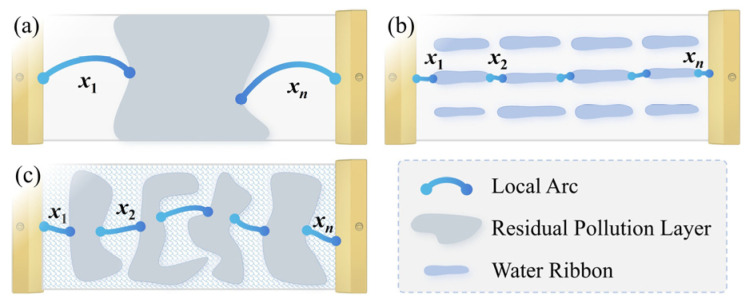
Static Obenaus model for (**a**) hydrophilic surface, (**b**) hydrophobic surface, and (**c**) hydrophilic/hydrophobic alternating surface.

The schematic representations of the static pollution flashover models for the three surface types are shown in Figure 16. For the purely hydrophilic surface, the flashover path is typically composed of one to two dominant dry-band arcs (Figure S3), i.e., *n*_philic_ ≈ 1–2. In contrast, for the alternating surface exhibiting the multi-arcs effect, the number of arcs (*n*_as_) is significantly higher and is strongly influenced by the distribution of hydrophobic interfaces.

According to Equation (16), the pollution flashover voltage of the hydrophilic/hydrophobic alternating surface (*U*_as_) can be estimated as

(17) $$U_{\mathrm{as}}=0.84\cdot n_{\mathrm{as}}+U_{\mathrm{Philic}}$$

Based on the analysis in Section 4.2, under ideal conditions, the number of multi-arcs (*n*_as_) on the alternating surface would be equal to the number of hydrophobic interfaces (*n*_phobic_). However, during actual discharge, the pollution layer temperature and current density change significantly after arc initiation. Adjacent dry bands may merge, leading to *n*_a_ < *n*_phobic_. Conversely, more than one dry band may form above a single hydrophobic interface, resulting in *n*_as_ > *n*_phobic_. Therefore, the number of multi-arcs is not solely determined by the number of hydrophobic interfaces, but is directly influenced by their spatial distribution.

**Figure 17:**
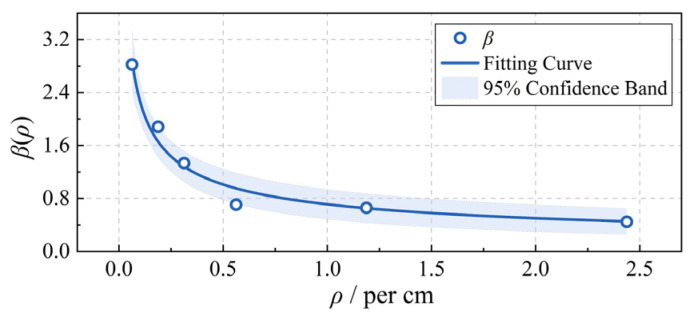
Correction coefficient of the multi-arc number versus alternating density.

To account for this deviation, a correction coefficient *β*(*ρ*), related to the alternating density ρ, is introduced to modify *n*_as_. By combining the experimental flashover voltage data obtained in Section 3 with the corresponding number of hydrophobic interfaces and substituting into Equation (17), the following empirical relationship can be derived (Figure 17):

(18) $$\left\{\begin{array}{l} n_{\mathrm{as}}=\beta (\rho )\cdot n_{\mathrm{phobic}}\\ \beta (\rho )=0.71\rho^{-0.51},\;\;\;\;s=0.5 \end{array}\right.$$

It can be observed that, as the alternating density (*ρ*) increases, the number of multi-arcs does not grow linearly with the number of hydrophobic interfaces, but gradually approaches saturation. Taking the W4 sample as an example, when both the hydrophobic and adjacent hydrophilic interfaces are 4 mm wide, the correction coefficient *β* decreases to 0.45. This indicates that two adjacent hydrophobic interfaces become electrically equivalent to a single dry band during discharge. This result is consistent with the macroscopic trend shown in Figure 6, where *U*_50%_ gradually saturates with increasing alternating density.

Under the condition of a fixed hydrophobic area fraction of 50%, the proposed static pollution flashover model incorporating the corrected number of multi-arcs enables the estimation of the flashover performance of embedded alternating surfaces with different alternating densities. The model therefore provides theoretical guidance for the structural optimization of embedded hybrid insulators, including the design of groove position, width, and spacing.

## 6. Conclusions

(1) A mechanical interlocking concept is introduced into the field of hybrid insulators. A manufacturing strategy is proposed in which concentric grooves are milled at the green-body stage and subsequently filled with silicone rubber. To evaluate the outdoor insulation performance of the resulting hydrophilic/hydrophobic alternating surfaces, a series of samples with a fixed hydrophobic area fraction of 50% are designed. Artificial pollution tests show that as the hydrophobic interfaces are increasingly segmented and the number of hydrophilic/hydrophobic interfaces increases, *U*_50%_ exhibits a saturating upward trend. When 39 interfaces are distributed within a 16 cm creepage distance, the pollution flashover voltage exceeds that of a fully hydrophobic surface by 12.4%.
(2) Combined high-speed imaging and infrared thermography reveal the electro–thermal coupled discharge mechanism of alternating surfaces. The flashover process can be divided into three stages: (Stage I) widespread point discharges occur at the hydrophilic/hydrophobic interfaces, accompanied by non-uniform evaporation and dry-band initiation above hydrophobic regions; (Stage II) discontinuous mm-scale dry-band arcs develop over discrete hydrophobic regions, forming a stable multi-arcs discharge channel; (Stage III) the multi-arcs merge and lead to complete flashover. The non-uniform conductivity distribution results in electric field concentration at the interfaces and Joule heating concentration above hydrophobic regions, which induces multiple coexisting dry bands. This mechanism fundamentally explains why the pollution flashover voltage is maintained or even enhanced.
(3) By introducing an additional source term into the Obenaus model, a static pollution flashover model applicable to hydrophilic/hydrophobic alternating surfaces is established. The model captures the discharge channel characterized by multi-arcs in series with the residual pollution layer on hydrophilic regions prior to flashover. Based on experimental data, a negative power-law relationship between the number of multi-arcs and the interface density is identified, enabling quantitative estimation of the pollution flashover voltage. The model further provides guidance for optimizing the spatial distribution of hydrophobic interfaces in embedded hybrid insulators (e.g., groove position and spacing at the green-body stage), allowing superior pollution flashover performance to be achieved even with a limited hydrophobic area fraction (≤50%), while maintaining structural durability.

## Figures and Tables

**Table 1:** Sample configurations used in this study.

ID	Width of Hydrophobic Interface (*w*)/mm	Number of Hydrophobic Interfaces (*n*_phobic_)	Alternating Density (*ρ*)/cm^−1^	Hydrophobic Area Fraction (*s*)
Philic	/	/	/	0
W4	4	20	2.44	0.5
W8	8	10	1.19	0.5
W16	16	5	0.56	0.5
W26.7	26.7	3	0.31	0.5
W40	40	2	0.19	0.5
W80	80	1	0.06	0.5
Phobic	160	/	/	1

## Data Availability

The original contributions presented in this study are included in the article, further inquiries can be directed to the corresponding author.

## Supplementary Materials

The following supporting information can be downloaded at https://www.mdpi.com/article/10.3390/polym18080904/s1, Figure S1: High-speed photography and infrared thermography of the pollution flashover process on a purely hydrophilic surface. Red strips denote the locations of the hydrophobic interfaces; Figure S2: High-speed photography and infrared thermography of the pollution flashover process on a purely hydrophobic surface. Red strips denote the locations of the hydrophobic interfaces; Figure S3: High-speed image sequence of arc development on the purely hydrophilic surface before flashover. The blue curve represents the leakage current magnitude during the corresponding half-cycle; Figure S4: High-speed image sequence of arc development on the purely hydrophobic surface before flashover. The blue curve represents the leakage current magnitude during the corresponding half-cycle.
