# Supplementary material for: Pollution Flashover Characteristics of Hydrophilic/Hydrophobic Alternating Surfaces for Insulator Hybridization
Source: Polymers (Basel). 2026 Apr 8;18(8):904. doi: 10.3390/polym18080904 (PMC13120617; doi:10.3390/polym18080904)
Supplement: Supplementary file 1 [file polymers-18-00904-s001.zip › polymers-4212565-supplementary.pdf]

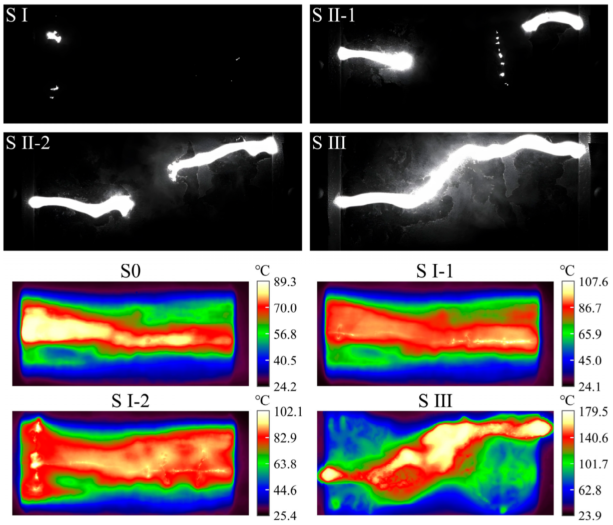

Figure S1. High-speed photography and infrared thermography of the pollution flashover process on a purely hydrophilic surface. Red strips denote the locations of the hydrophobic interfaces.

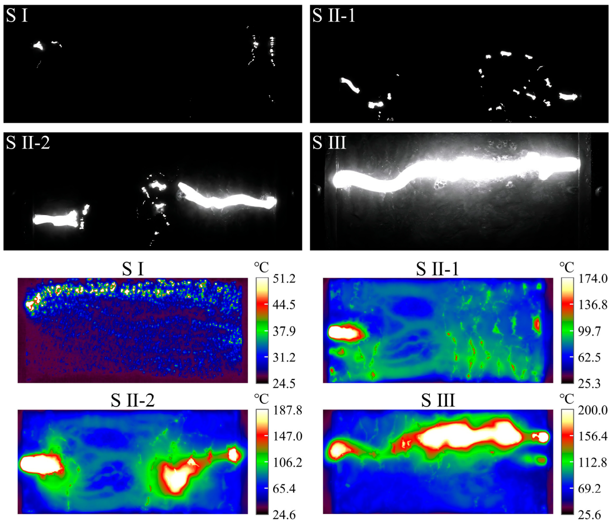

Figure S2. High-speed photography and infrared thermography of the pollution flashover process on a purely hydrophobic surface. Red strips denote the locations of the hydrophobic interfaces.

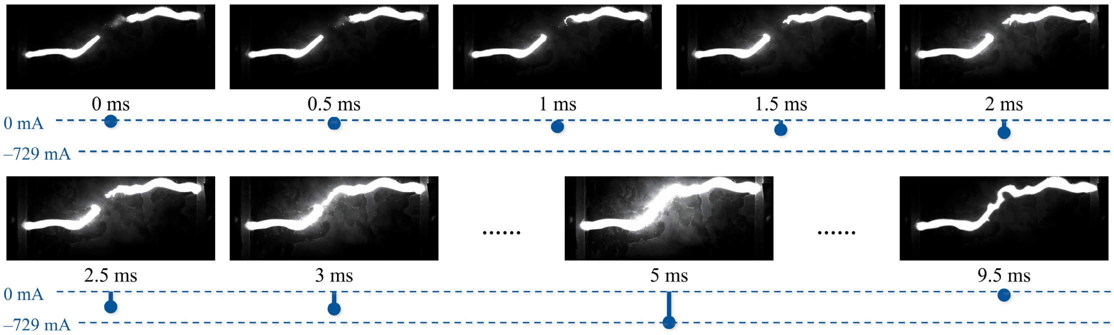

Figure S3. High-speed image sequence of arc development on the purely hydrophilic surface before flashover. The blue curve represents the leakage current magnitude during the corresponding half-cycle.

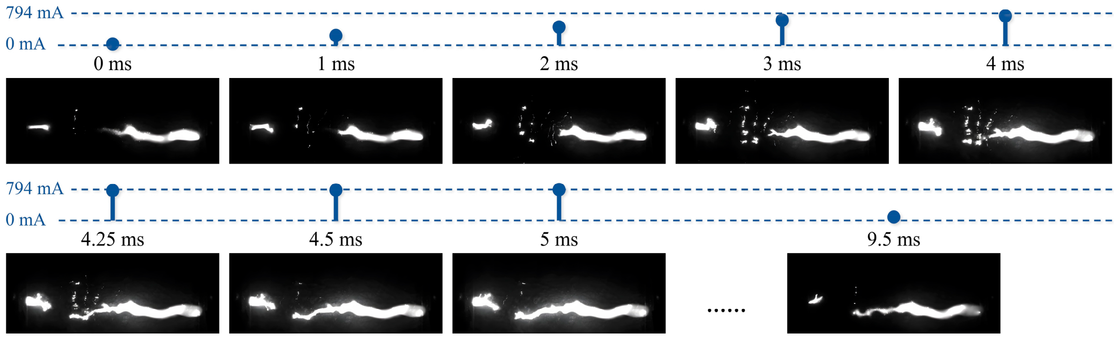

Figure S4. High-speed image sequence of arc development on the purely hydrophobic surface before flashover. The blue curve represents the leakage current magnitude during the corresponding half-cycle.
